# Supplementary material for: Thalamocortical control of cell-type specificity drives circuits for processing whisker-related information in mouse barrel cortex
Source: Nat Commun. 2023 Sep 28;14:6077. doi: 10.1038/s41467-023-41749-x (PMC10539368; doi:10.1038/s41467-023-41749-x)
Supplement: Supplementary file 1 — Supplementary Information [file 41467_2023_41749_MOESM1_ESM.pdf]

Supplementary Information

Supplementary Methods

Supplementary Figures: Supplementary Figs 1-11

Supplementary Methods

Clustering of L4 single-cell RNA sequencing (scRNA-seq) data

Layer 4 scRNA-seq data were obtained from two scRNA-seq datasets from mouse somatosensory cortex[1,2] (P4: GSM5277845 [https://www.ncbi.nlm.nih.gov/geo/query/acc.cgi?acc=GSM5277845]; P7: GSE204759 [https://www.ncbi.nlm.nih.gov/geo/query/acc.cgi?acc=GSE204759]) and analyzed in R with the Seurat package. For P7 data, L4 cells were obtained using annotations included in the dataset. For P4 data, preprocessing filtering (200<nfeatures<4500, mito%<10), and clustering (npcs=40, resolution=0.5) was performed to identity the L4 population. Unsupervised clustering was performed on merged P4 and P7 data following a Harmony-implemented correction. Hollow and septa signature scores were calculated as above and Kmeans clustering (k=4) performed using these scores. Differential gene expression testing was performed using the Wilcoxon Rank Sum test with Bonferroni correction.

Identification of potential ligand-target interactions in the thalamocortical pathway

Given the importance of TCA innervation for barrel formation, a method (NicheNet, v1.1.1) to predict ligand-target links based on the transcriptomes of interacting cell populations was employed to identify potential molecular interactions in the TCA-barrel pathway[3]. Two sections containing VPM were obtained from P7 control brains for spatial transcriptomics profiling, as described above. VPM barcodes (n=105) were filtered from the dataset using *Slc6a4* expression and combined with L4 barcodes, obtained in our previous analysis of the barrel cortex, as the inputs for NicheNet modeling. Here, Celsr3 cKO barcodes were also included as a 'condition' group to help highlight potentially important target molecules (absolute log2FC>0.25, vs control). VPM ligands were restricted to the best 150 candidates and the top quartile of potential ligand-target interactions were highlighted for the analysis.

DiI labeling of thalamocortical projections

To label thalamocortical projections, post-fixed brains (P6-P7) were embedded in 6% (w/v) low-melt agarose in PBS, then sectioned caudorostrally in the coronal plane using a vibratome. Once the appropriate level was reached, a single DiI crystal (Invitrogen) was inserted into VPM, then incubated at 4°C for several weeks in 1% PFA. Sections (40 μm) were collected through S1 and counterstained with DAPI.

Supplementary Figures

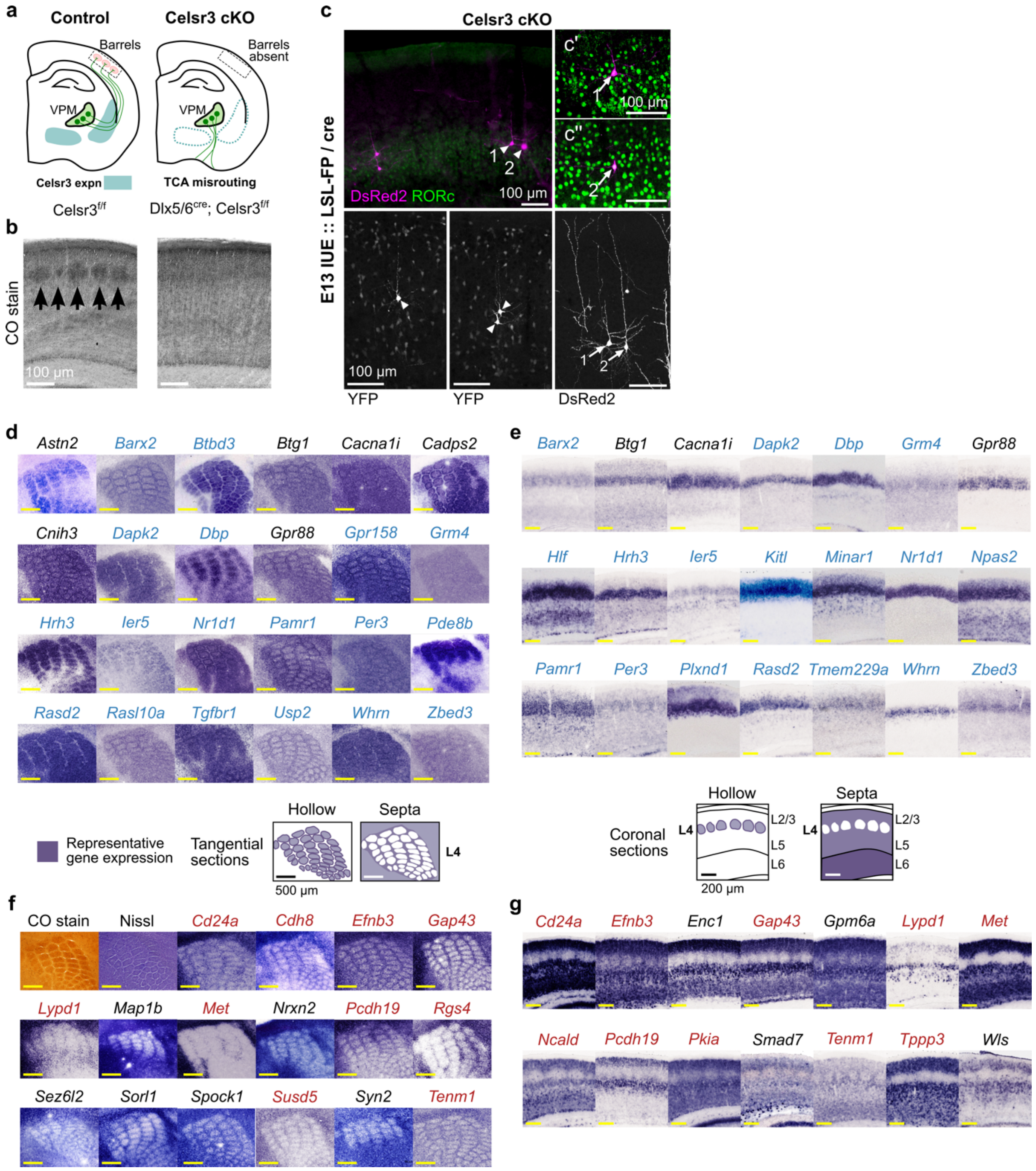

Supplementary Figure 1. Reciprocal hollow- and septa-related gene expression patterns in mouse barrel cortex. **a**, Schematic illustrating the pathfinding defect of TCAs in Celsr3 cKOs, which arises due to targeted knockout in the ventral telencephalon using the Dlx5/6$^{IRES\text{-}Cre}$ driver. As a result, TCAs do not innervate the cortex. **b**, Cytochrome oxidase (CO) staining. **c**, Morphology of L4 neurons in Celsr3cKOs. Neurons were sparsely labeled with a Cre-dependent lox-STOP-lox-fluorescent protein (LSL-FP) construct using *in utero* electoration (IUE). Top, Celsr3cKO neurons were positioned in L4 and positive for RORc (c′-c′′). Bottom, confocal images showing examples of pyramidal morphology of Celsr3cKO neurons.

Some neurons displayed signs of retarded dendritic development. Scale bars, 100 μm. (**d-g**) ISH of screened candidate markers showing distinct whisker-related patterns in tangential (**d** and **f**) and coronal (**e** and **g**) sections through mouse somatosensory cortex at P6-P7. Schema of representative ISH expression patterns are shown in the center panels. Scale bars: tangential, 500 μm; coronal, 200 μm. (**d-g**) Hollow-related markers were expressed in periodic clusters within L4, similar to the patterns of Nissl and CO staining (shown in **d**) that align with whisker compartments. Several genes were strongly expressed within the barrel hollows (eg.*Pde8b, Rasd2, Whrn*) while others displayed concentrated expression around the inner edge of each hollow (eg.*Barx2, Nr1d1, Usp2*). (**f** and **g**) Screened septa markers exhibited a reciprocal pattern of expression between barrel hollows. In coronal sections, septal expression is broadly observed across cortical layers, but notably restricted from L4 barrel cortex resulting in a periodic net-like pattern between hollows. Candidate markers that were used to guide clustering of L4 transcriptomic data are colored in blue (hollow) and red (septa).

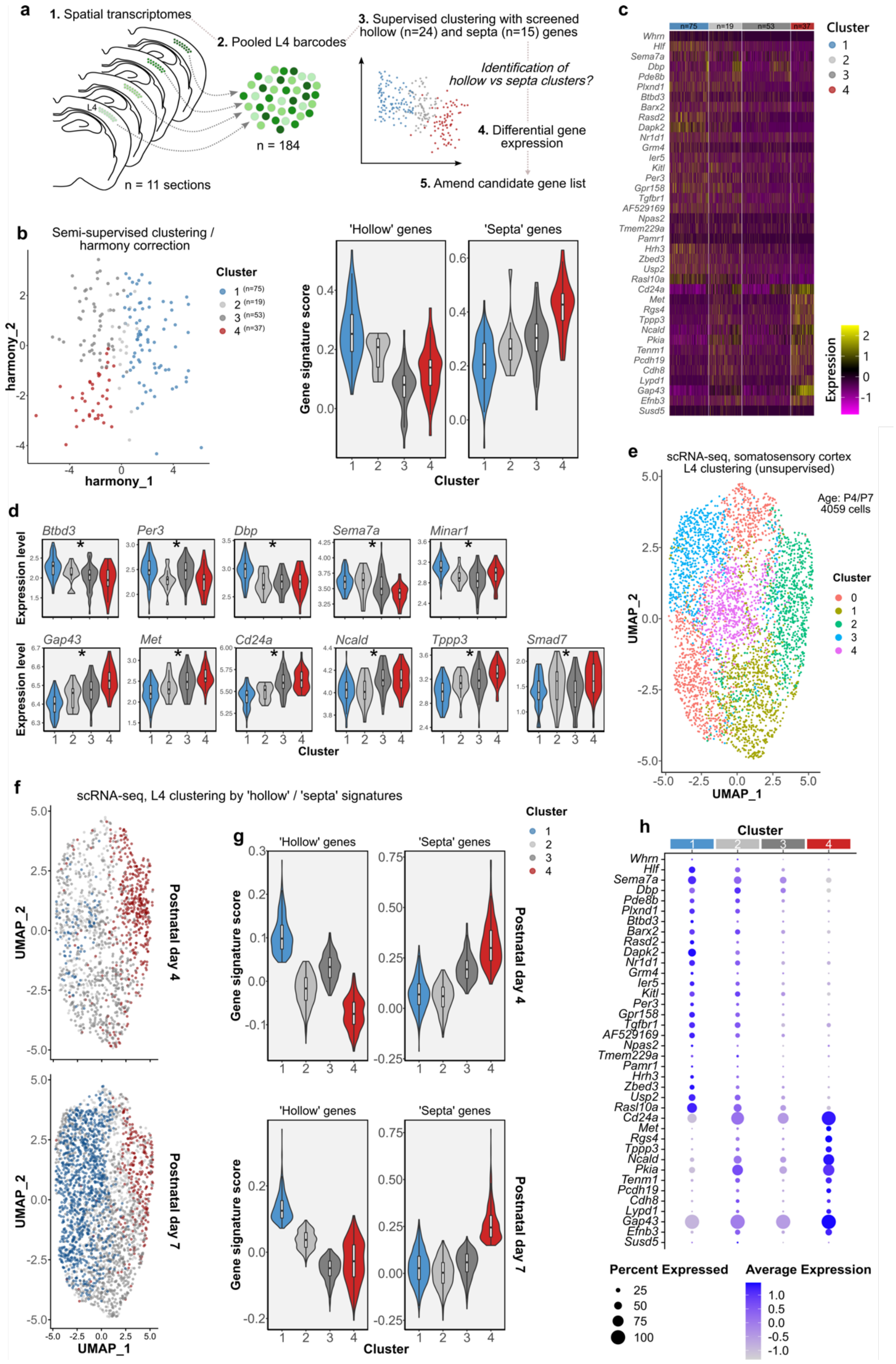

Supplementary Figure 2. Clustering analysis of L4 transcriptomic data. **a**, L4 spatial barcodes were isolated and semi-supervised clustering performed using 38 screened candidate hollow/septa genes. **b**, Following batch correction, four clusters

(left) were identified showing differential expression of marker genes as assessed by hollow and septa gene signature scores (right). Cluster 1 (blue, n=75) displayed high hollow and low septa scores, likely representing L4 spots that sample a higher number of spiny stellate neurons. Cluster 4 displayed reciprocal low hollow and high septa scores, likely biased towards septa/pyramidal neurons (red, n=37). **c**, Heatmap displaying differential enrichment of hollow and septa markers among detected clusters. **d**, Cluster expression of representative screened hollow/septa markers used for clustering. Asterisks indicate * $p<0.05$, Cluster 1 (n=75) vs Cluster 4 (n=37), two-sided Wald test. **e**, Unsupervised clustering of L4 cells from P4 and P7 mouse somatosensory cortex scRNA-seq datasets. **f**, Clustering of P4 (top, Cluster 1, n=80; Cluster 2, n=271; Cluster 3, n=594; Cluster 4, n=413) and P7 (bottom, Cluster 1, n=1023; Cluster 2, n= 659; Cluster 3, n=776; Cluster 4, n=243) L4 cells using hollow/septa scores, visualized via the UMAP space shown in (**e**). Differential gene expression was performed on Clusters 1 (putative hollow, blue) and 4 (putative septa, red), which displayed inverse hollow/septa gene signature scores. **g**, Hollow/septa gene signature scores for the 4 clusters shown in (**f**). **h**, Dot plot displaying enrichment of 38 marker genes within each L4 cluster obtained from the scRNA-seq datasets. Graphical plots display violin and boxplot distributions for each cluster. For all boxplots, the interquartile range (IQR) is plotted with the median as the center line and no outliers displayed. Whiskers represent minima and maxima within 1.5 x IQR from the first and third quartiles.

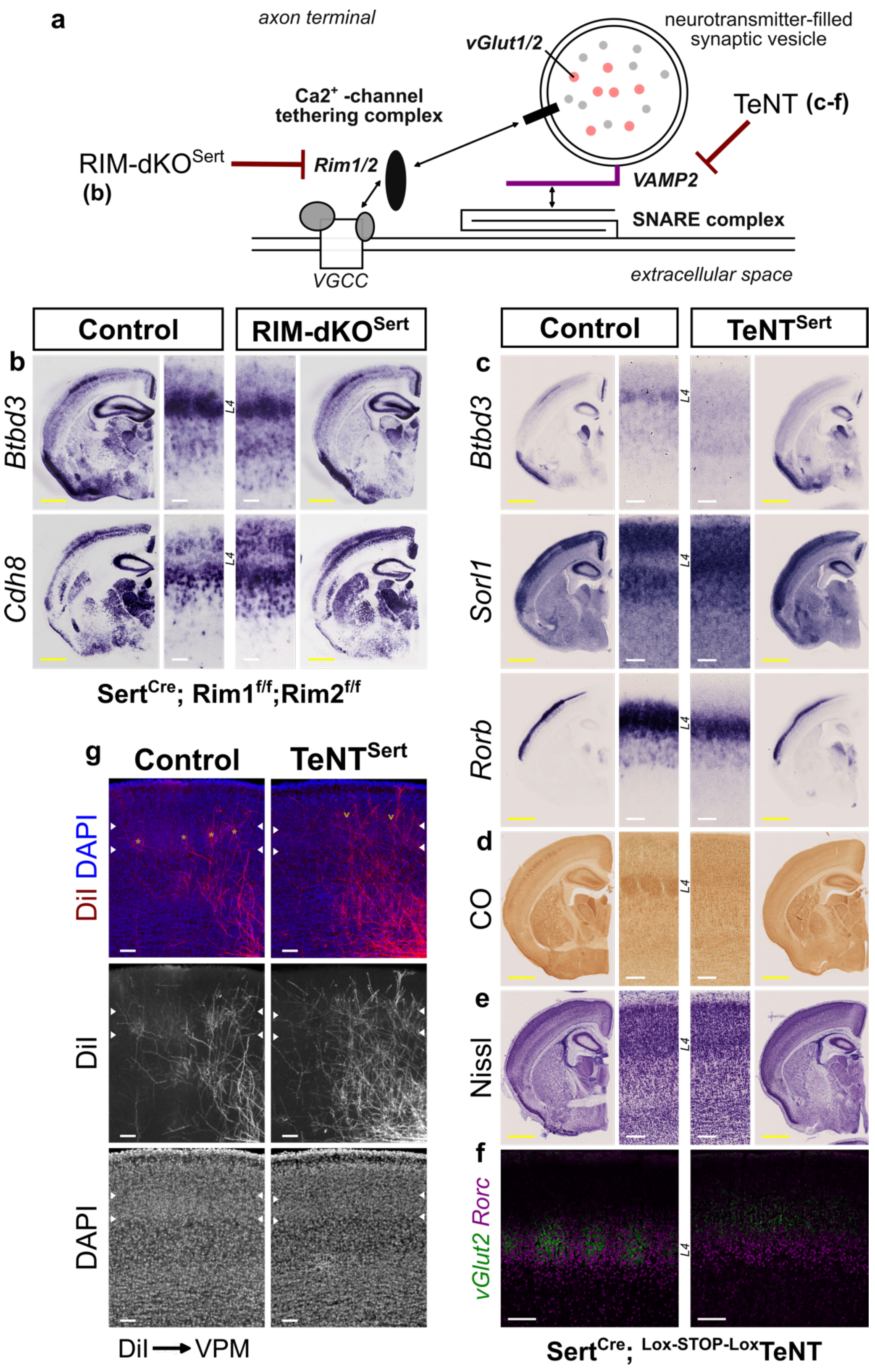

Supplementary Figure 3. Genetic perturbation of thalamocortical activity and effect on barrel gene expression. a, Schema depicting two manipulations utilized for disruption of TCA neurotransmission, acting at the level of synaptic vesicular release. In Rim1/2dKO^{Sert} animals (indicated by (b) and results are in b), calcium-dependent synaptic release is impaired[4].

Tetanus toxin overexpression in TCAs (Sert^{Cre}; TeNT^{Tg}) prevents the SNARE-mediated exocytosis of synaptic vesicles, strongly impairing neurotransmission[5,6] (indicated as (c-f) and results are in c-f). **b**, the size and gene expression of barrel cortex was comparable in control and Rim1/2dKO brains. **c**, L4 barrel gene expression was severely impacted in Sert^{Cre}; TeNT^{Tg} mice (*Btbd3* downregulation, *Sorl1* upregulation) and was associated with a lack of barrel structures seen by cytochrome oxidase (CO) (**d**) and Nissl staining (**e**). **f**, vGlut2 clusters are not seen in L4 of Sert^{Cre}; TeNT^{Tg} mice. **g**, DiI placement into VPM resulted in labeled axons in S1 cortex, however, in Sert^{Cre}; TeNT^{Tg} mice these did not form distinct clusters in L4 as seen in controls. Scale bars, 1 mm (yellow), 100 µm (white).

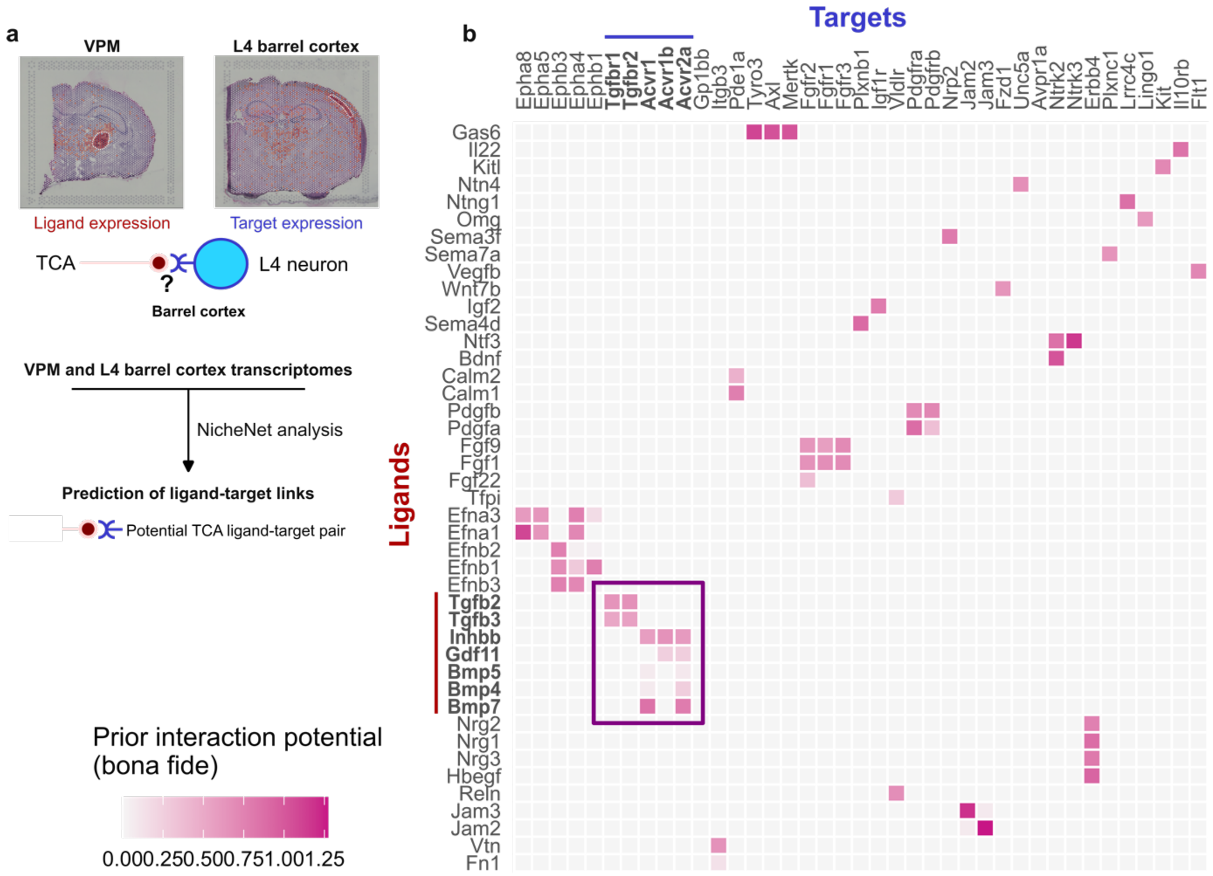

Supplementary Figure 4. Identification of potential ligand-target interactions within the thalamocortical-barrel pathway. **a**, 10X Visium data were filtered using Slc6a4 expression or L4 markers, to obtain P6 transcriptomes for VPM and barrel cortex, respectively. These were used as input for a ligand-receptor analysis using NicheNet[3]. **B**, Heatmap displaying the top quartile of potential interactions as determined by NicheNet analysis. Several ligand and receptors of the TGF-β family were identified (purple box). Source data are provided as a Source Data file.

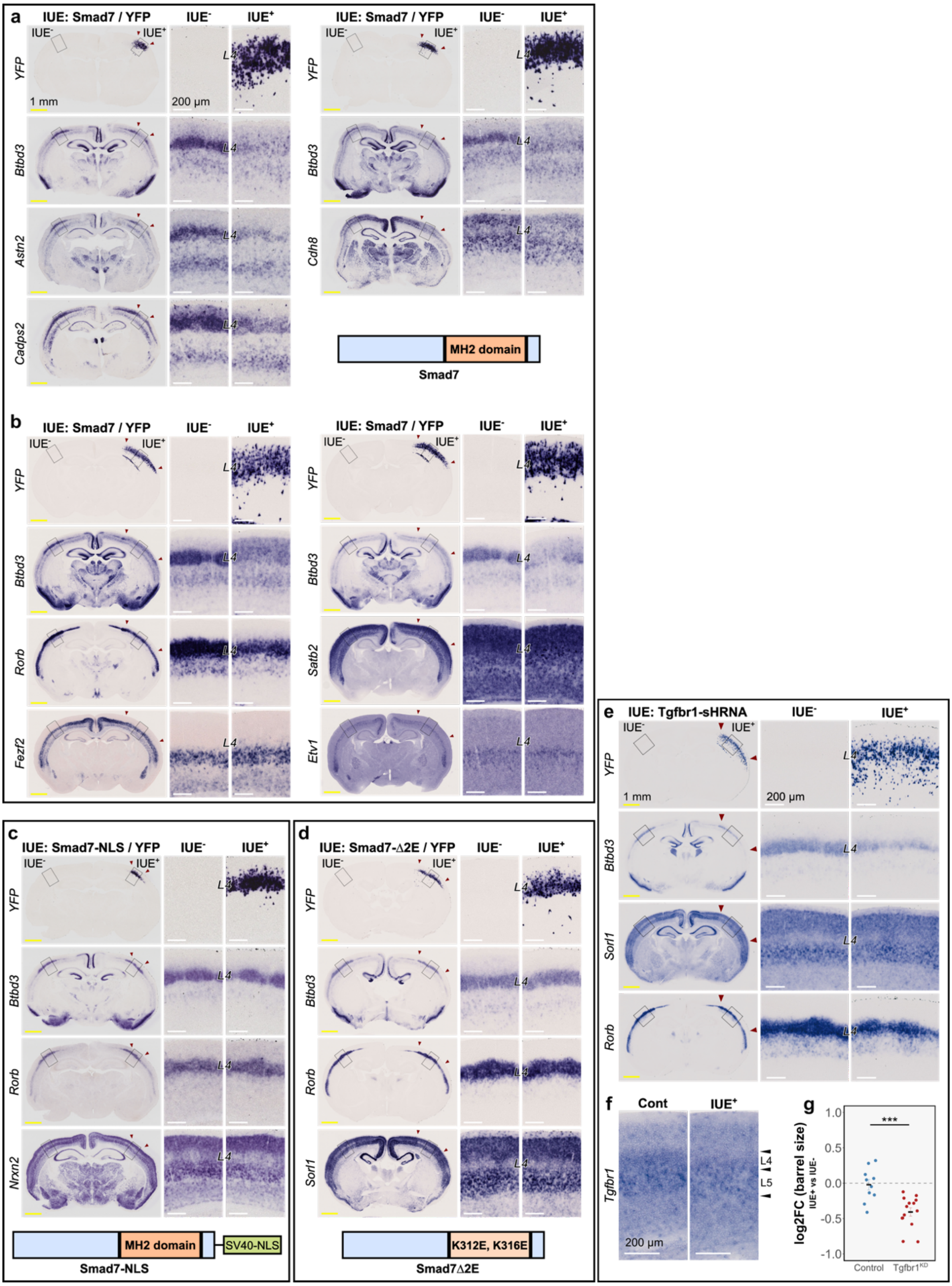

Supplementary Figure 5. Specific changes to barrel cortex with Smad7^{OE}. a, Images of adjacent sections from an example Smad7/EYFP electroporated brain at P7 (Smad7^{OE}) following ISH for representative hollow (*Btbd3*, *Astn2*, and *Cadps2*) and septa (*Cdh8*) genes. Respective down- and up-regulation of genes is seen only

within the area of electroporation, indicated by red arrowheads in YFP sections (IUE^+ hemisphere), compared to the non-electroporated side (IUE^- hemisphere). **b**, Normal positioning of cortical layer markers (*Rorb*, *Fezf2*, *Satb2*, and *Etv1*) with $Smad7^{OE}$. (**c-d**) Normal barrel gene expression with overexpression of Smad7 mutants. **c**, Nuclear-localized Smad7 (Smad7-NLS) and **d**, Tgfbr1-binding mutant ($Smad7^{K132E,K316E}$) displayed unaltered expression of barrel gene markers in electroporated regions. **e**, shRNA knockdown of *Tgfbr1* by IUE ($Tgfbr1^{KD}$). In the transfected hemisphere, $Tgfbr1^{KD}$ reduced the size and expression of hollow markers (*Btbd3*) in barrel structures, with reciprocal upregulation of septa (*Sorl1*) markers. **f**, Knockdown was confirmed with *Tgfbr1* ISH, reducing expression intensity by ~35±4% (n=5) compared to the unelectroporated cortex. Representative knockdown of *Tgfbr1* expression is shown. In this example, a large area of electroporation resulted in downregulation of *Tgfbr1* in both L4 and L5. **g**, Quantification of changes in barrel size with $Tgfbr1^{KD}$. For each brain, the change in barrel area was calculated from Btbd3-labeled sections (IUE^+ versus IUE^-). Here, differences are expressed as log2FoldChange, with $Tgfbr1^{KD}$ compared to a non-targeting shRNA control. Asterisks indicate p=0.0007, two-sided Welch's t-test (Control, n=10; Tgfbr1KD, n=14 brains. Group means ± SEM are displayed. Scale bars, 1 mm (yellow) and 200 µm (white). Source data are provided as a Source Data file.

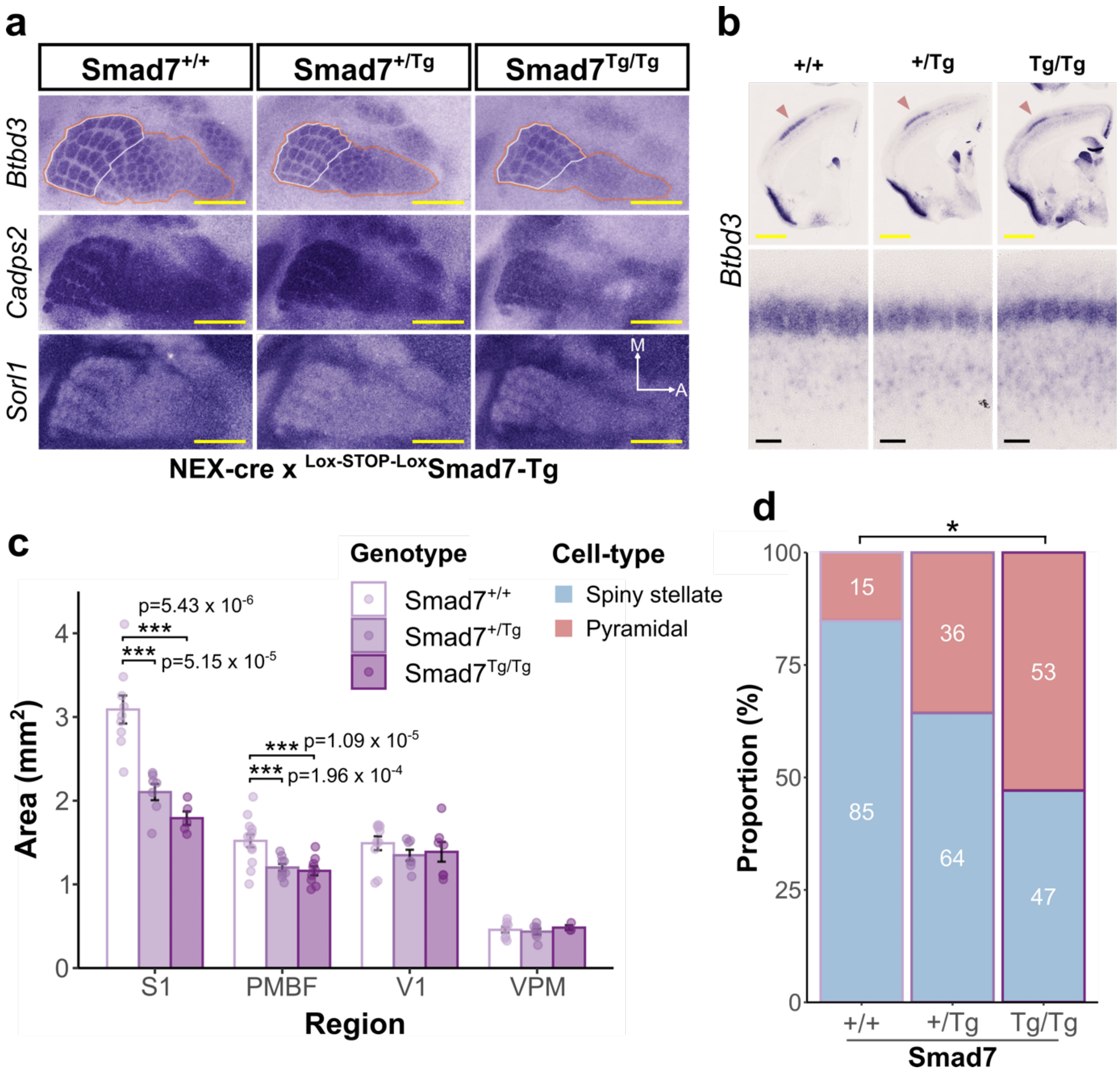

Supplementary Figure 6. Alteration to barrel structures in Smad7 transgenic animals. a, in NEX$^{Cre;}$ Smad7^{Tg} mice, smaller barrel structures were observed in tangential sections (P7) stained using riboprobes against hollow (*Btbd3, Cadps2*) and septa (*Sorl1*) markers. Anterior barrels were also less defined compared to controls. **b,** Appearance of barrels within anterior S1 in coronal sections. **c,** Quantification of size of barrel structures in Smad7^{Tg} mice. Areas of S1 and PMBF (posteromedial barrel field) were measured from regions in **a**, outlined by orange and white, respectively. Data are presented as group means ± SEM. Asterisks indicate *** $p < 0.001$, linear mixed-effects model with Kenward-roger approximation and Tukey adjustment for multiple comparisons. For S1-PMBF-V1: Smad7$^{+/+}$, n=13; Smad7$^{+/Tg}$, n=10; Smad7$^{Tg/Tg}$, n=9 brains. For VPM: Smad7$^{+/+}$, n=8 Smad7$^{+/Tg}$, n=7; Smad7$^{Tg/Tg}$, n=3 brains. **d**, Quantification of spiny stellate versus pyramidal morphology in barrel cortex of NEX^{Cre}; Smad7^{Tg} mice. Morphology was assessed using a whole-cell patch

setup. Asterisk indicates $p<0.05$, generalized linear mixed-effects model with parametric bootstrap test, $Smad7^{+/+}$ versus $Smad7^{Tg/Tg}$ ($Smad7^{+/+}$, n=33; $Smad7^{+/Tg}$, n= 14; $Smad7^{Tg/Tg}$, n=17 cells). Scale bars, 1mm (yellow) and 100 μm (black). Source data are provided as a Source Data file.

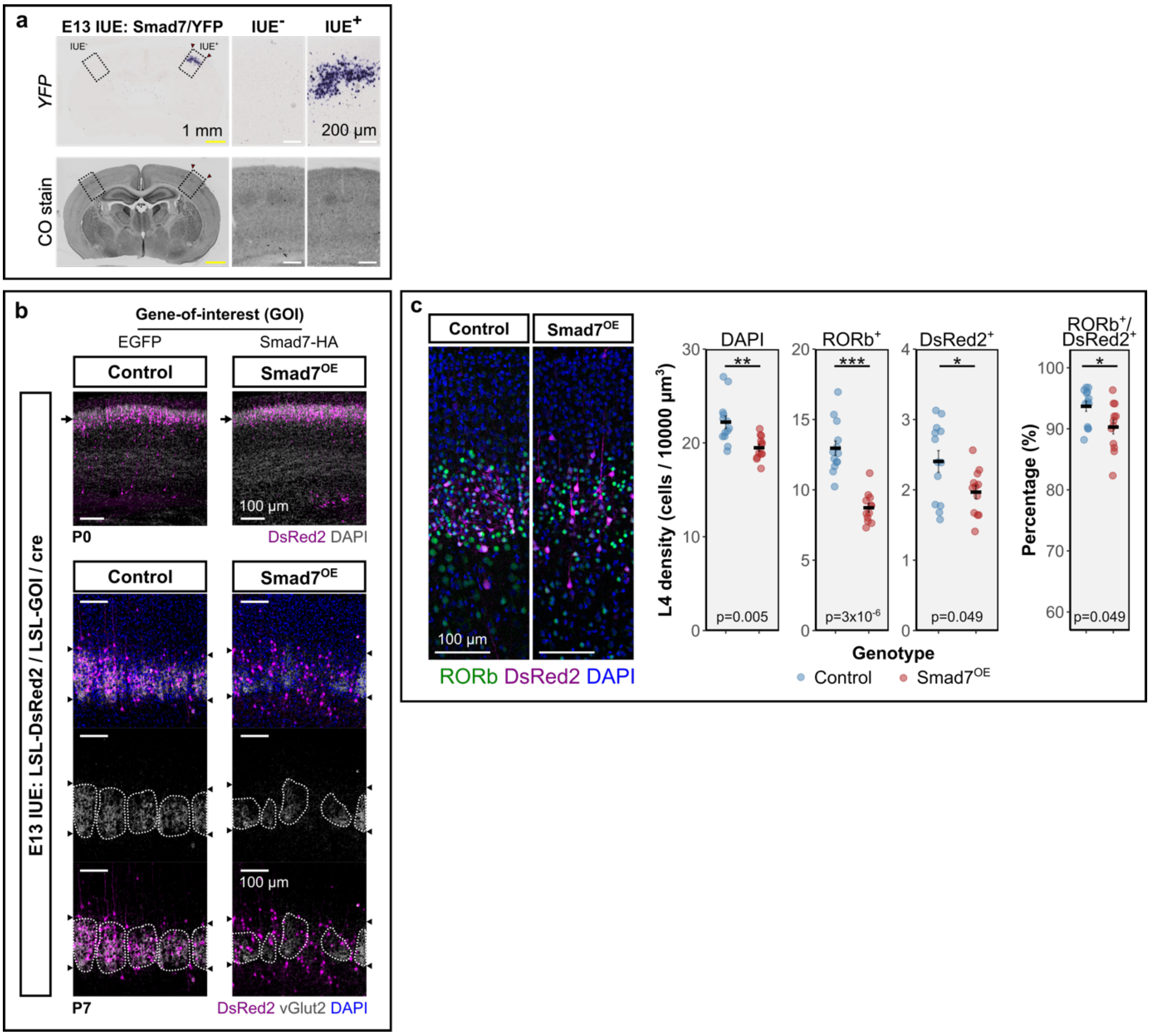

Supplementary Figure 7. Additional cytoarchitectural characterization of Smad7^{OE} brains. **a**, Size of barrel clusters stained with cytochrome oxidase (CO) were smaller with Smad7^{OE}. Scale bars in **a**: 1 mm (yellow) and 200 µm (white). **b**, Control and Smad7^{OE} neurons were transfected using IUE with Cre-dependent (LSL=LoxP-STOP-LoxP) plasmids: DsRed2 plus gene-of-interest (GOI). Coronal sections are shown at P0 (top) and P7 (bottom). Arrows indicate the position of neurons in the cortical plate at P0. Locations of vGlut2 clusters at P7 are outlined with white dashes, while arrowheads indicate the position of L4. Scale bars, 100 µm. **c**, Quantification of L4 cell density in Smad7^{OE} brains at P7. The density of neurons was calculated from DAPI and Rorb^{+} immunostaining situated in L4. Data are presented as group means ± SEM. Asterisks indicate * $p<0.05$, ** $p<0.01$, *** $p<0.001$; two-sided Welch's t-test with multiple comparison correction using the Holm method (n=12-13 sections from 4 brains per genotype). Scale bars, 100 µm. Source data are provided as a Source Data file.

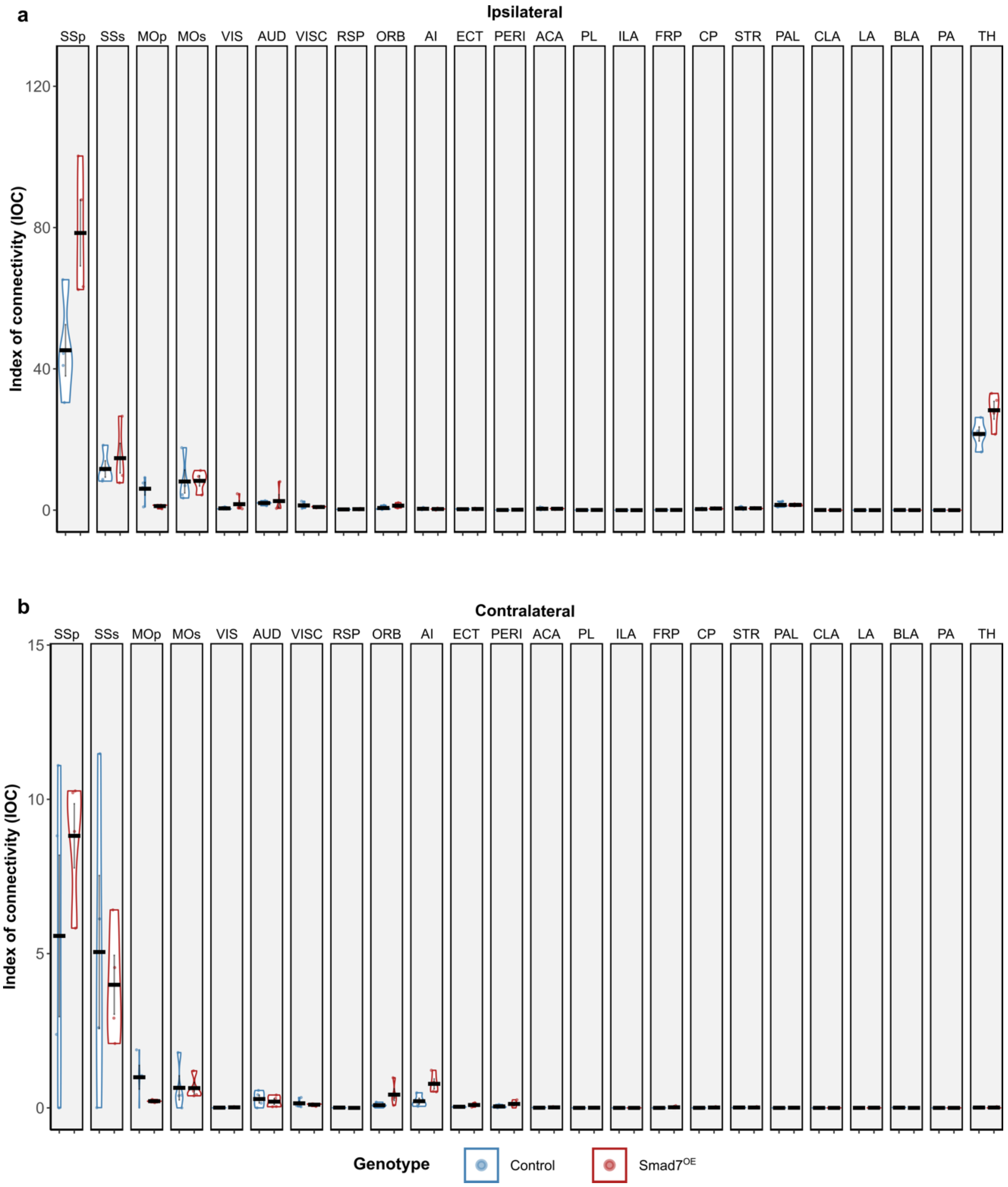

Supplementary Figure 8. Rabies virus tracing of presynaptic neurons in Smad7^{OE} brains. Index of connectivities (IOC) for **a**, ipsilateral and **b**, contralateral brain regions after rabies tracing of Control and Smad7^{OE} starter neurons. Violin plots show distributions along with group means ± SEM (n=4 control, n=4 Smad7^{OE} brains). Abbreviations: SSp, primary somatosensory cortex; SSs, secondary somatosensory cortex; MOp, primary motor cortex; Mos, secondary motor cortex; VIS, visual areas; AUD: auditory areas; VISC, visceral areas; RSP, retrosplenial area; ORB, orbital area; AI, agranular insular area; ECT, ectorhinal area; PERI, perirhinal area;

ACA, anterior cingulate area; PL, prelimbic area; ILA, infralimbic area; FRP, frontal pole; CP, caudoputamen; STR, striatum; PAL, pallidum; CLA, claustrum; LA, lateral amygdalar nucleus; BLA, basolateral amygdalar nucleus; PA, posterior amygdalar nucleus; TH, thalamus. Source data are provided as a Source Data file.

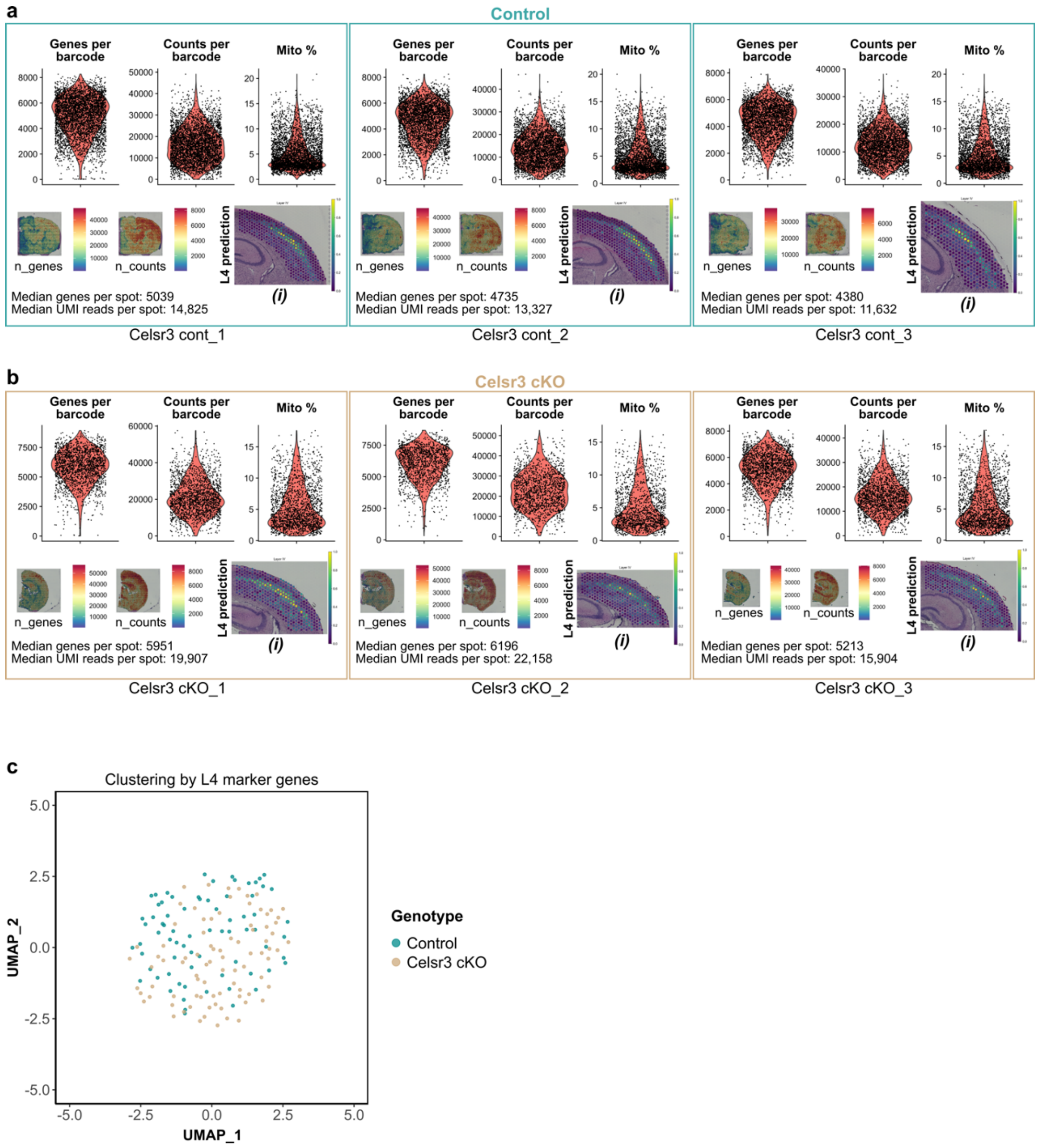

Supplementary Figure 9. Quality control metrics for Visium spatial transcriptome profiling of Celsr3 cKO somatosensory cortex. Spatial transcriptomics performed on **a**, Control and **b**, Celsr3 cKO sections containing primary somatosensory cortex (n=3 per genotype). (Top) Violin plots displaying total number of genes, transcript number, and mitochondrial percentage per spatial barcode. (Bottom) Distribution of total number of genes and total number of transcripts for spatial barcodes found across the profiled section. (i) Analyses were performed on spatial barcodes situated over somatosensory cortex. The probability of each barcode being of ‘L4’ identity, as predicted by scRNAseq/Tangram integration, is indicated by the color scale. **c**, The overall clustering of Control and Celsr3 cKO L4 barcodes using layer 4 marker genes was observed within a similar UMAP space.

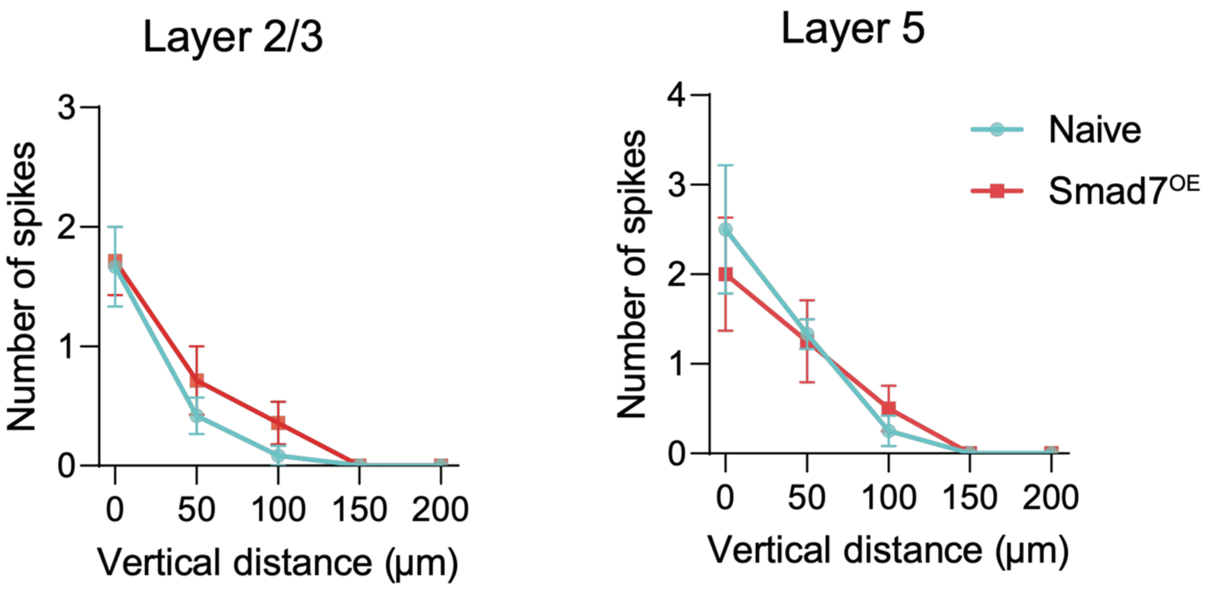

Supplementary Figure 10. Spatial resolution of photostimulation of cortical neurons. The number (mean ± SEM) of spikes evoked by photostimulation in recorded L2/3 cells (left) and L5 cells (right) plotted against the vertical distance from the center of laser spot to the soma of the recorded cells. Spikes were recorded extracellularly from naive (cyan) and Smad7^{OE} neurons (red). The laser spot was shifted vertically through the center of the soma, resulting in the generation of action potentials in neurons whose cell bodies were generally located within ~100 µm from the center of the illuminated spot. No significant differences were found in the number of spikes between the naive and Smad7^{OE} neurons at distances of 0, 50, 100, 150 or 200 µm (t-test). The number of cells in layer 2/3: n=6 naïve, n=7 Smad7^{OE}; in layer 5: n=6 naïve, n=6 Smad7^{OE} neurons. Source data are provided as a Source Data file.

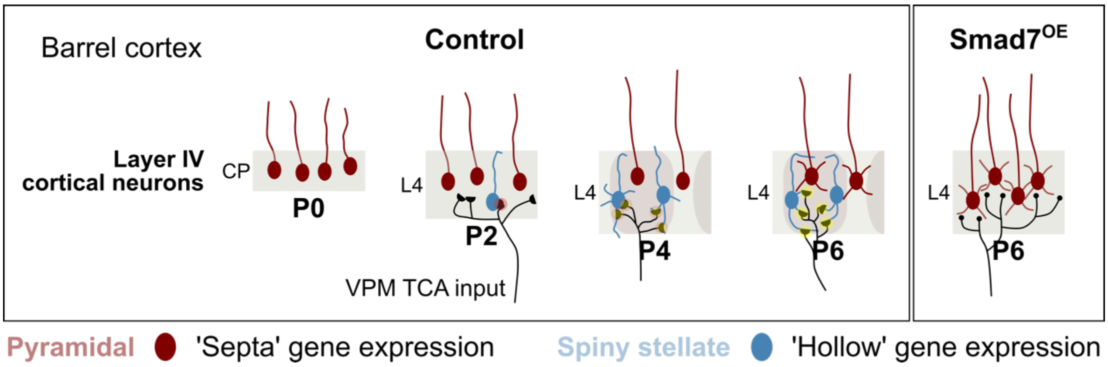

Supplementary Figure 11. Summary schematic illustrating the relationship between thalamocortical axons and developing L4 cortical neurons in barrel cortex. Normal development is shown on the left. Smad7 overexpression (right) impairs acquisition of spiny stellate fate and results in alterations to barrel connectivity and processing of whisker-related information. Abbreviations: CP, cortical plate; VPM: ventroposteromedial nucleus; TCA, thalamocortical axon; L4, layer 4; P{N}, postnatal age N; OE, overexpression.

Supplementary References

1. Di Bella, D. J. *et al.* Molecular logic of cellular diversification in the mouse cerebral cortex. *Nature* **595**, 554–559 (2021).
2. Yuan, W. *et al.* Temporally divergent regulatory mechanisms govern neuronal diversification and maturation in the mouse and marmoset neocortex. *Nat Neurosci* **25**, 1049–1058 (2022).
3. Browaeys, R., Saelens, W. & Saeys, Y. NicheNet: modeling intercellular communication by linking ligands to target genes. *Nat Methods* **17**, 159–162 (2020).
4. Narboux-Nême, N. *et al.* Neurotransmitter Release at the Thalamocortical Synapse Instructs Barrel Formation But Not Axon Patterning in the Somatosensory Cortex. *J. Neurosci.* **32**, 6183–6196 (2012).
5. Sando, R. *et al.* Assembly of Excitatory Synapses in the Absence of Glutamatergic Neurotransmission. *Neuron* **94**, 312-321.e3 (2017).
6. Sakamoto, M. *et al.* Continuous postnatal neurogenesis contributes to formation of the olfactory bulb neural circuits and flexible olfactory associative learning. *J Neurosci* **34**, 5788–5799 (2014).
