# Supplementary material for: Thalamocortical control of cell-type specificity drives circuits for processing whisker-related information in mouse barrel cortex
Source: Nat Commun. 2023 Sep 28;14:6077. doi: 10.1038/s41467-023-41749-x (PMC10539368; doi:10.1038/s41467-023-41749-x)
Supplement: Supplementary file 3 — Description of Additional Supplementary Files [file 41467_2023_41749_MOESM3_ESM.pdf]

Description of Additional Supplementary Files

File name: Supplementary Data 1
Description: Summary of genes used for L4 clustering in barrel cortex.

File name: Supplementary Data 2
Description: Summary of differential gene expression analyses using transcriptomics data.

File name: Supplementary Data 3
Description: List of DNA constructs, sequences, reagents, and resources.
