# Supplementary material for: Thalamocortical control of cell-type specificity drives circuits for processing whisker-related information in mouse barrel cortex
Source: Nat Commun. 2023 Sep 28;14:6077. doi: 10.1038/s41467-023-41749-x (PMC10539368; doi:10.1038/s41467-023-41749-x)
Supplement: Supplementary file 7 — Reporting Summary [file 41467_2023_41749_MOESM7_ESM.pdf]

nature portfolio

Corresponding author(s): Tomomi Shimogori

Last updated by author(s): Aug 17, 2023

Reporting Summary

Nature Portfolio wishes to improve the reproducibility of the work that we publish. This form provides structure for consistency and transparency in reporting. For further information on Nature Portfolio policies, see our Editorial Policies and the Editorial Policy Checklist.

Statistics

For all statistical analyses, confirm that the following items are present in the figure legend, table legend, main text, or Methods section.

n/a	Confirmed	
☐	☒	The exact sample size (*n*) for each experimental group/condition, given as a discrete number and unit of measurement
☐	☒	A statement on whether measurements were taken from distinct samples or whether the same sample was measured repeatedly
☐	☒	The statistical test(s) used AND whether they are one- or two-sided<br>*Only common tests should be described solely by name; describe more complex techniques in the Methods section.*
☐	☒	A description of all covariates tested
☐	☒	A description of any assumptions or corrections, such as tests of normality and adjustment for multiple comparisons
☐	☒	A full description of the statistical parameters including central tendency (e.g. means) or other basic estimates (e.g. regression coefficient) AND variation (e.g. standard deviation) or associated estimates of uncertainty (e.g. confidence intervals)
☐	☒	For null hypothesis testing, the test statistic (e.g. *F*, *t*, *r*) with confidence intervals, effect sizes, degrees of freedom and *P* value noted<br>*Give P values as exact values whenever suitable.*
☒	☐	For Bayesian analysis, information on the choice of priors and Markov chain Monte Carlo settings
☒	☐	For hierarchical and complex designs, identification of the appropriate level for tests and full reporting of outcomes
☒	☐	Estimates of effect sizes (e.g. Cohen's *d*, Pearson's *r*), indicating how they were calculated

Our web collection on statistics for biologists contains articles on many of the points above.

Software and code

Policy information about availability of computer code

Data collection	NDP.scan (Hamamatsu, v3.2.17), FLUOVIEW (FV31S-SW, v2.3.2.169, Olympus), BZ-X700 Viewer (v01.03.00.01, Keyence), PCO.sdk (PCO, v1.14.0), and custom scripts in Matlab
Data analysis	FIJI-ImageJ (NIH, v1.53j); Space Ranger (10x Genomics, v1.3.1); Mini Analysis Program (Synaptosoft); CellProfiler (Stirling et al. 2021, v4.2.5); MATLAB (Mathworks, R2018b v9.5) with open source code: AMaSiNe (Song et al. 2014, v1.0); R (v4.1.0) with R libraries: Seurat (Hao et al. 2021, v4), Harmony (Korsunsky et al. 2019, v1.0), NicheNet (Browaeys et al. 2020, v1.1.1), DESeq2 (Love et al. 2014, v1.34.0); Python (v3.8) with libraries: Tangram (Biancalani et al. 2021, v1.0.3), SimpleITK (v1.2.4), numpy (v1.18.1), opencv2 (v3.4.2.16), scikit-image (v0.16.2), scanpy (v1.9.1), scipy (v1.5.2); original code generated in this study has been deposited in the CBS repository system {url}.

For manuscripts utilizing custom algorithms or software that are central to the research but not yet described in published literature, software must be made available to editors and reviewers. We strongly encourage code deposition in a community repository (e.g. GitHub). See the Nature Portfolio guidelines for submitting code & software for further information.

Data

Policy information about availability of data

All manuscripts must include a data availability statement. This statement should provide the following information, where applicable:

- Accession codes, unique identifiers, or web links for publicly available datasets
- A description of any restrictions on data availability
- For clinical datasets or third party data, please ensure that the statement adheres to our policy

Spatial transcriptomics data have been deposited in the CBS repository system {url} and are publicly available as of the date of publication.
Other data generated and/or analyzed in this study are available from the corresponding author upon reasonable request.
This paper also analyzes existing, publicly available data: Allen Mouse Brain Adult Reference Atlas (CCFv3; https://atlas.brain-map.org/), scRNA-seq datasets (accession codes: GSM5277845, GSE204759).

Human research participants

Policy information about studies involving human research participants and Sex and Gender in Research.

Reporting on sex and gender	N/A
Population characteristics	N/A
Recruitment	N/A
Ethics oversight	N/A

Note that full information on the approval of the study protocol must also be provided in the manuscript.

Field-specific reporting

Please select the one below that is the best fit for your research. If you are not sure, read the appropriate sections before making your selection.

☒ Life sciences ☐ Behavioural & social sciences ☐ Ecological, evolutionary & environmental sciences

For a reference copy of the document with all sections, see nature.com/documents/nr-reporting-summary-flat.pdf

Life sciences study design

All studies must disclose on these points even when the disclosure is negative.

Sample size	Statistical methods were not used to determine sample size. We estimated sample sizes based on experience from previous investigations and similar experiments described in the literature (PMID: 21799210, PMID: 31591398, PMID: 31591398, PMID: 35289744, PMID: 24828045, PMID: 34707291). For our behavioral paradigm, a minimum of 6 mice per group was used to achieve power >90% at a significance level of 0.05 (PMID: 23026377).
Data exclusions	In behavioral experiments, mice were excluded from analysis if they exhibited less than 4 interactions with either texture during the trial. This threshold was used to increase the likelihood that novelty detection was due to discrimination between two textures, and not resulting from an innate bias. For rabies viral tracing, animals in which starter neurons were found outside the barrel cortex were excluded. For imaging experiments, animals in which the electroporated area was found outside the barrel cortex were excluded. For retrograde CTB labeling, animals in which injections missed the target region were excluded.
Replication	Experiments were performed using multiple independent litters (>3), with the exception of some spatial transcriptomics experiments. Due to limited resources, only one control and one cKO brain was collected for comparison, using serial sections (n=3) as replicates. All attempts at replication were successful.
Randomization	Mice were allocated to their experimental group based on genotype.
Blinding	Where possible, blinding was performed during data collection and analysis. Celsr3 cKO animals were visibly smaller in size compared to control littermates, making it hard to perform blinding during collection.

Reporting for specific materials, systems and methods

We require information from authors about some types of materials, experimental systems and methods used in many studies. Here, indicate whether each material, system or method listed is relevant to your study. If you are not sure if a list item applies to your research, read the appropriate section before selecting a response.

Materials & experimental systems

n/a	Involved in the study
☐	☒ Antibodies
☒	☐ Eukaryotic cell lines
☒	☐ Palaeontology and archaeology
☐	☒ Animals and other organisms
☒	☐ Clinical data
☒	☐ Dual use research of concern

Methods

n/a	Involved in the study
☒	☐ ChIP-seq
☒	☐ Flow cytometry
☒	☐ MRI-based neuroimaging

Antibodies

Antibodies used

Rabbit anti-GFP, polyclonal (MBL International, Cat# 598, RRID:AB_591819)
Rat anti-GFP (clone GF090R), monoclonal (Nacalai Tesque, Cat# 04404-84, RRID:AB_10013361)
Rabbit anti-DsRed, polyclonal (Takara Bio, Cat# 632496, RRID:AB_10013483)
Rat anti-HA (clone 3F10), monoclonal (Roche, Cat# 11867431001, RRID:AB_390919)
Rabbit anti-beta subunit Cholera Toxin, polyclonal (Abcam, Cat# ab34992, RRID:AB_726859)
Guinea pig anti-vGlut2, polyclonal (Synaptic Systems, Cat# 135 404, RRID:AB_887884)
Mouse anti-ROR common (clone H3925), monoclonal (Cosmo Bio, Cat# PP-H3925-00, RRID:AB_605116)
Mouse anti-RORbeta (clone N7927), monoclonal (Cosmo Bio, Cat# PP-N7927-00, RRID:AB_1964364)
Rabbit anti-Smad7, polyclonal (Abcam, Cat# ab216428, RRID: AB_2889839)
Goat anti-rabbit IgG (H+L) Highly Cross-Adsorbed Secondary Antibody, Alexa Fluor 488 (Thermo Fisher Scientific, Cat# A-11034, RRID:AB_2576217)
Goat anti-rabbit IgG (H+L) Highly Cross-Adsorbed Secondary Antibody, Alexa Fluor 594 (Thermo Fisher Scientific, Cat# A-11037, RRID:AB_2534095)
Goat anti-rat IgG (H+L) Cross-Adsorbed Secondary Antibody, Alexa Fluor 488 (Thermo Fisher Scientific, Cat# A-11006, RRID:AB_2534074)
Goat anti-rat IgG (H+L) Cross-Adsorbed Secondary Antibody, Alexa Fluor 647 (Thermo Fisher Scientific, Cat# A-21247, RRID:AB_141778)
Cy3 AffiniPure Donkey Anti-Guinea Pig IgG (H+L) (Jackson ImmunoResearch, Cat# 706-165-148, RRID: AB_2340460)
Alexa Fluor 647 AffiniPure Donkey Anti-Guinea Pig IgG (H+L) Jackson ImmunoResearch, Cat# 706-605-148, RRID: AB_2340476)
Sheep Anti-Digoxigenin Fab fragments Antibody, POD Conjugated (Roche, Cat# 11207733910, RRID:AB_514500)

Validation

The antibodies have been tested and validated (Western blotting, cell lines, and/or section staining) by the corresponding company, which can be found on the manufacturer's website, along with an extensive list of citations. Where applicable, antibodies were chosen based on the literature and their reported application in mouse neuronal tissue: anti-DsRed (PMID: 34707291, 19477955, PMID: 21925217, PMID: 28781050, PMID: 29551417); anti-CTB (PMID: 33782621, PMID: 33589834, PMID: 35320722), anti-HA (PMID: 29358753, PMID:36229429), anti-GFP (PMID: 28641108, PMID: 37219072, PMID: 26193445, PMID: 31239441, PMID: 25821910), anti-vGlut2 (KO validated, PMID: 35063073, PMID: 34296997), anti-Smad7 (Western blot, tissue staining), anti-RORc (PMID: 29779941, PMID: 30639110), anti-RORbeta (PMID: 30057116, PMID: 31395862)

Animals and other research organisms

Policy information about studies involving animals; ARRIVE guidelines recommended for reporting animal research, and Sex and Gender in Research

Laboratory animals

Mice were group-housed with ambient temperature set to 24 ± 2°C and relative humidity at 55 ± 10%. ICR, C57BL/6J, and transgenic mouse strains were used in this study. Mice were used at both developmental (P0 - P18) and adult stages (>P42). Transgenic mice used include: Celsr3 conditional knockouts obtained by crossing female Celsr3(f/f) mice (RRID:MGI:3579160) with male Celsr3(f/+);Dlx5/6-Cre+/- mice (RRID:MGI:3795743). Male Sert-cre mice (B6.129(Cg)-Slc6a4tm1(cre)Xz/J; RRID:IMSR_JAX:014554)81 were bred with female mice from a Cre-inducible tetanus toxin (TeNT) strain (B6;129S6-Gt(ROSA)26Sortm1.1(CAG-EGFP/TeNT)Imayo; RRID:IMSR_RBRC05154) to obtain heterozygote TeNT-Tg/- animals, with Cre-positive animals compared to Cre-negative controls. Fixed Rim1/2dKO (Sert-cre x Rim1f/fRim2f/f ; RRID:MGI:4939901) brain samples (P8) were obtained from Dr Patricia Gaspar. For widefield calcium imaging, Thy1-GCaMP6f (RRID: IMSR_JAX:024276) male mice were mated with female C57BL/6 mice and in utero electroporation was performed on pregnant dams. Conditional Smad7-Tg were generated using homologous recombination in mouse ES ES cells and backcrossed to a C57BL/6 background. F1 mice were bred with Nex-cre mice for cortical overexpression of the Smad7 transgene.

Wild animals

This study did not use wild animals.

Reporting on sex

Mice of either sex were used for this study.

Field-collected samples

This study did not involve field-collected samples.

Ethics oversight

All procedures were performed in accordance with protocols approved by the Institutional Animal Care and Use Committees of

Ethics oversight	RIKEN Wako and Kobe branches, and the National Institute for Physiological Sciences. All experiments conformed to RIKEN regulations for scientific research.

Note that full information on the approval of the study protocol must also be provided in the manuscript.
